# A novel mutation of *ABHD5* gene in a Chanarin Dorfman patient with unusual dermatological findings

**DOI:** 10.1186/s12944-019-1181-6

**Published:** 2019-12-28

**Authors:** Ali Haydar Eskiocak, Sara Missaglia, Laura Moro, Murat Durdu, Daniela Tavian

**Affiliations:** 1 Department of Dermatology, Baskent University Faculty of Medicine, Adana Hospital, Adana, Turkey; 2 Laboratory of Cellular Biochemistry and Molecular Biology, CRIBENS, Catholic University of the Sacred Heart, pz Buonarroti 30, 20145 Milan, Italy; 3 Department of Psychology, Catholic University of the Sacred Heart, Largo Gemelli 1, 20123 Milan, Italy; 4 Department of Pharmaceutical Sciences, University of Piemonte Orientale, Novara, Italy

**Keywords:** Chanarin-Dorfman syndrome, Ichthyosis, Lipid disorder, Liver involvement, Pityriasis rubra pilaris, Hyperlipidemia

## Abstract

**Background:**

Chanarin Dorfman Syndrome (CDS) is a rare autosomal recessive disorder characterized by the multisytemic accumulation of neutral lipids inside the cytoplasmic lipid droplets. This condition is caused by mutations in the abhydrolase domain containing 5 gene (*ABHD5*). In CDS the skin involvement is the prevalent and always observed clinical feature, consisting of a non-bullous congenital ichthyosiform erythroderma (NCIE). Moreover, a variable involvement of the liver and neuromuscular system can be also observed. In this report, we aimed to perform the clinical and genetic characterization of a patient affected by CDS with atypical dermatological findings, considering this rare inborn error of neutral lipid metabolism.

**Methods:**

Genomic DNA samples obtained from patient and his parents were used to perform the sequencing of the *ABHD5* exons and their intron/exon boundaries. Bioinformatic analyses were performed to investigate the possible effect of the identified mutation on protein structure.

**Results:**

Here we present the case of a 29-year-old male patient with CDS, who, for long time, has been misdiagnosed as pityriasis rubra pilaris (PRP). He has a history of increasing hyperlipidemia; hepatomegaly associated with hepatosteatosis was also detected. *ABHD5* molecular analysis revealed a novel missense mutation, the c.811G > A (p.G271R). Bioinformatic investigations showed that the variant has a deleterious effect on ABHD5 function, probably causing an incorrect folding of the mutant protein.

**Conclusions:**

These results highlihts the importance of genetic testing for *ABHD5* in unresolved cases of patients presenting unusual skin lesions, that resemble PRP, associated with a history of hyperlipidemia and nonalcoholic fatty liver.

## Background

**Fig. 1:**
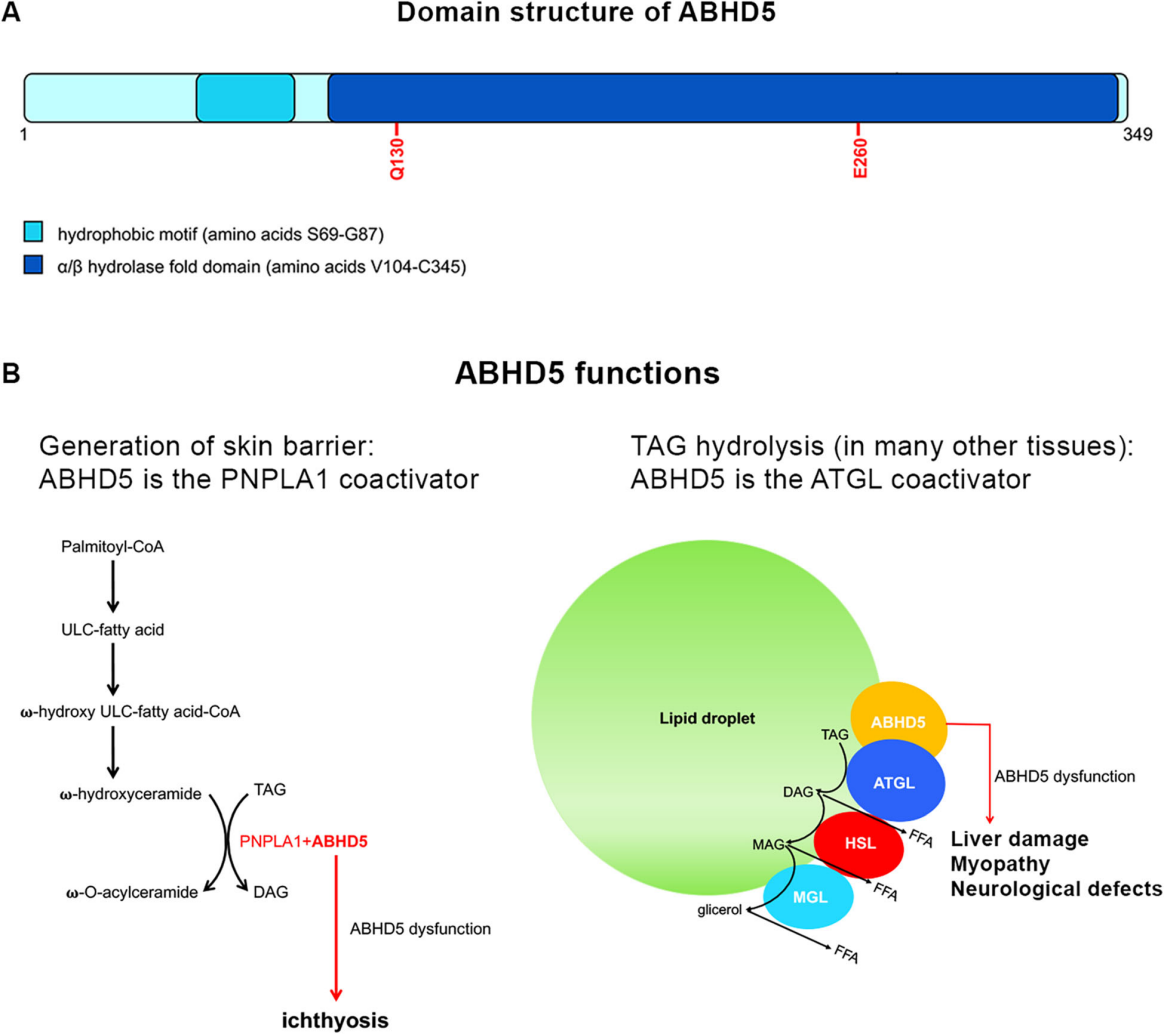
ABHD5 protein structure and function. Schematic representation of ABHD5 protein (**a**). The ABHD5 protein is characterized by two functional domains: a LD-binding site (hydrophobic domain) and an α/βhydrolase domain that includes two amino acid residues involved in ATGL and perilipin interaction (Q130 and E260). ABHD5 as coactivator of PNPLA1 and ATGL (**b**). During the formation of the skin barrier (keratinocytes), ABHD5 activates PNPLA1 determining the production of ὠ–O-acylceramide. In liver, muscle and many other tissues (including skin), ABHD5 acts as ATGL coactivator, inducing TAG hydrolysis

Chanarin Dorfman syndrome (CDS; MIM: 275630) is a rare neutral lipid storage disease, characterized by the intracellular accumulation of triacylglycerol (TG) in numerous tissues including skin, liver, skeletal muscle, eyes, ears, central nervous system and bone marrow [1]. The clinical diagnosis is based on detection of Jordans’ bodies (JBs), characteristic cytoplasmatic vacuoles in the granulocytes of patients [2]. To date, 150 cases have been described worldwide [3, 4]. Many of them have been reported from the Mediterranean and Middle-East region, especially Turkey, where consanguineous marriages are still common [5]. Mutations in *ABHD5* have been demonstrated as the responsible defect of CDS [6]. They cause the impairment of ABHD5 function, leading to an abnormal accumulation of TG stored into lipid droplets (LDs) in many tissues [7]_._ Indeed, ABHD5 acts as a coactivator for adipose triglyceride lipase (ATGL), which hydrolyzes the first fatty acid from TG [8]. Non bullous-congenital ichthyosiform erythroderma (NCIE) is the dominant feature of CDS, but additional clinical features can include liver damage, myopathy, neurosensory hearing loss, subcapsular cataracts, nystagmus, strabismus and mental retardation. It has been recently demontrated that NCIE is caused by the impairment of ABHD5 function which does not depend by ATGL lipase activity. Besides ATGL, ABHD5 is able to activate PNPLA1, which catalyzes the final step of ὠ–O-acylceramide production, an essential lipid for correct skin barrier formation in human keratinocytes [9] (Fig. 1).

Here we report one more CDS family where the affected patient presents a peculiar skin phenotype resenbling pityriasis rubra pilaris and, for this reason, he had been misdiagnosed for long time.

## Methods

### Histopathologic examination

A punch biopsy was fixed in formalin. The histologic sections for light microscopy were stained with haematoxylin and eosin.

Fresch EDTA-treated peripheral blood sample from patient, was centrifugated at 3300 g for 10 min. Buffy coat was collected, smeared onto slides glasses, dried and stained with May-Grünwald Giemsa (MGG) stain.

### Molecular analysis

To evaluate *ABHD5* exons sequences and their intron/exon boundaries, genomic DNAs of patient and his parents were amplified as previously reported [7]. All PCR products were purified using Nucleo Spin Extract kit (Mcherey-Nagel) and sequenced on 3730 DNA Analyzers by the BigDye® Terminator V1.1 Cycle Sequencing Kit (Applied Biosystems). Informed consent was obtained from all participants and analyses were performed in accordance with the Declaration of Helsinki principles.

### Bioinformatic investigation of *ABHD5* missense mutation

NCBI reference sequence of the human ABHD5 protein (NP_001342115.1) was used as template to evaluate possible effects of the identified missense mutation on ABHD5 function by ClustalW, PolyPhen and Mutation Tasting. ABHD5 wild-type and ABHD5 (G271R) 3D models were generated using I-Tasser, an in silico protein modeling tool.

## Results

### Case presentation

**Fig. 2:**
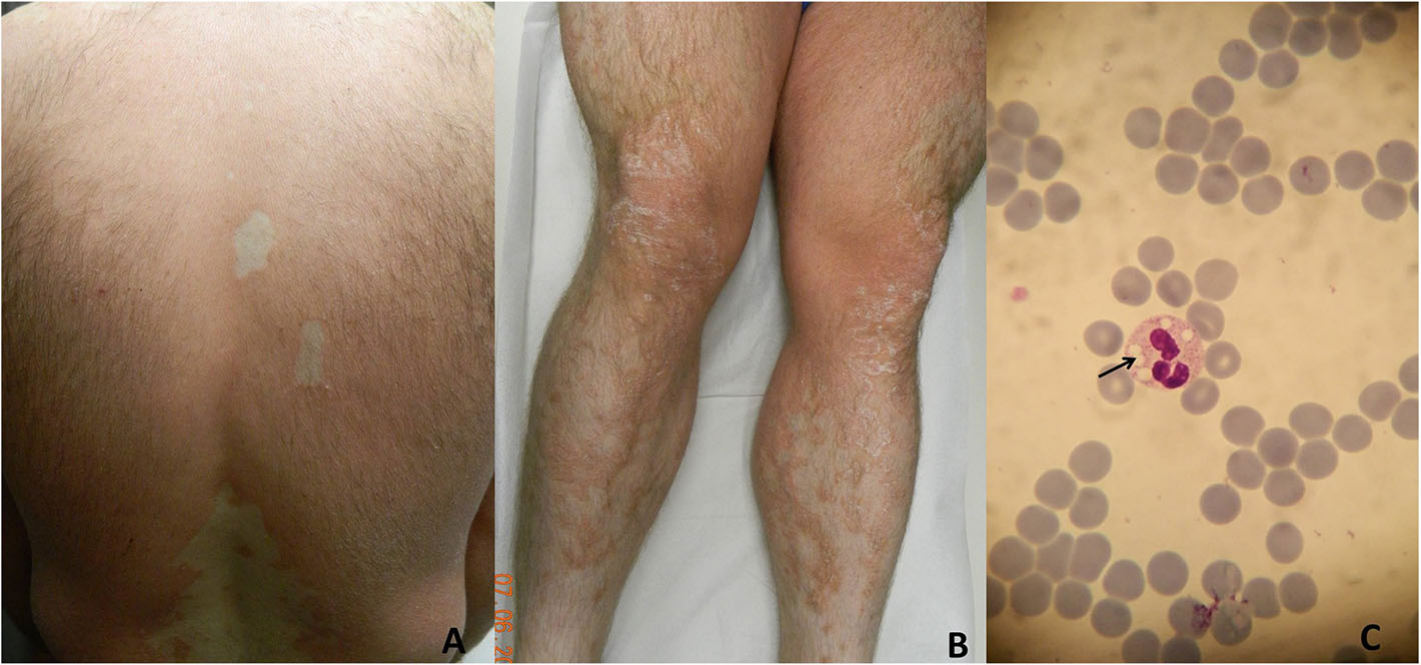
Clinical presentation of CDS patient. Erythematous and scaly lesions on back (**a**) and lower limbs (**b**) with uninvolved areas. Intracytoplasmic vacuoles (Jordans’bodies) in neutrophil of peripheral blood smear (**c**) (May-Grünwald-Giemsa X1000)

**Fig. 3:**
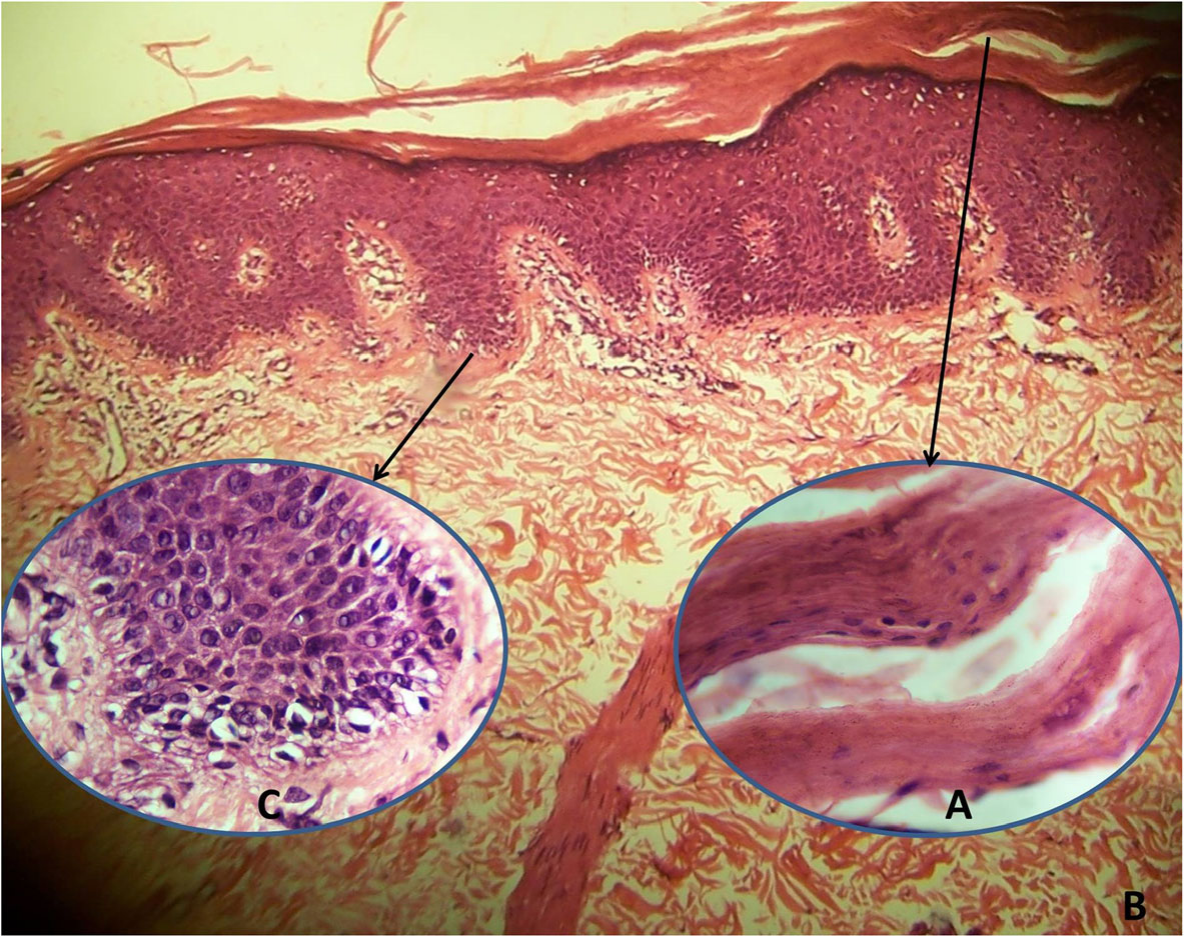
Histopathologic examination shows orthokeratosis, focal parakeratosis (**a**), flattening and merging of rete ridges (**b**), and vacuolar appearance in the cytoplasm of basal keratinocytes (**c**) (H&E, A and C × 1000; B × 100)

A-29-year old man was admitted to our outpatient clinic for erythematous scaly lesions that had been present since the age of 2 years. He had been previously biopsied twice and was diagnosed with pityriasis rubra pilaris. He received oral acitretin treatment for a long time but he did not respond to treatment. His parents were non-relatives and no one else in his family had a similar disease. Most recently, a dermatological examination performed in our clinic, revealed extensive erythematous patch and plaques on all parts of the body accompanied by fine scaling especially on lower limbs (Fig. 2a, b). The scaling was evident on border of the erythematous plaques. Strikingly, there were unaffected areas between the plaques on the back and the limbs. His face and scalp had seborrheic dermatitis-like mild erythema and fine scales. Ectropion, nail distrophy or palmoplantar involvement was not detected. Histopathologic examination of punch biopsy revealed orthohyperkeratosis (Fig. 3a), focal parakeratosis, flattening and merging of rete ridges and mild perivascular lymphocytic infiltration (Fig. 3b). Dilated follicular infundibulum with hyperkeratotic plug, a histopathologic feature of pityriasis rubra pilaris, was not detected. Notably, lipid vacuoles were observed in the cytoplasm of basal keratinocytes (Fig. 3c).

Muscle strength was normal and electromyographic (EMG) examination showed no signs of myopathy. Patient denied any hearing or visual problem. The mental status and neurologic examination were normal. Liver transaminases, creatine kinase (CK) and serum TG levels were elevated (alanine transaminase: 132 U/L, normal 5–35 U/L; aspartate transaminase: 83 U/L, normal 5–17 U/L; CK: 580 U/L, normal 22–200 U/L; TG: 381 mg/dl, normal 35–130 mg/dl). Abdominal ultrasonography was compatible with hepatomegaly associated with hepatosteatosis.

As our patient had a history of hyperlipidemia associated with hepatomegaly and his skin lesions got worse when serum triglyceride levels increased, CDS was suspected. Therefore, peripheral blood smear was performed and prominent intracytoplasmic vacuoles (JBs) were detected in neutrophils when stained with MGG (Fig. 2c).

### Genetic investigation

**Fig. 4:**
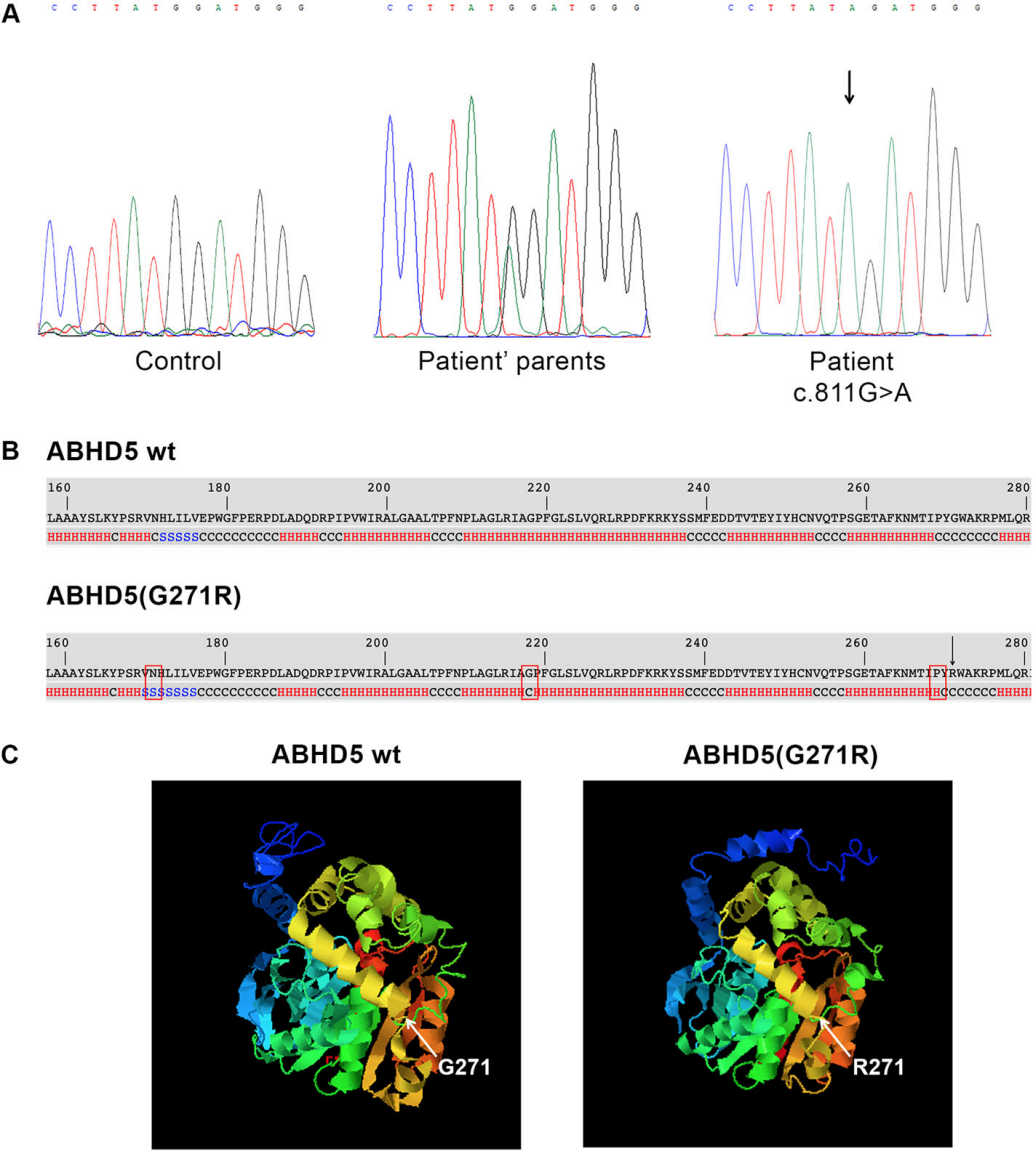
Molecular analysis of CDS patient and bioinformatic evaluation of the identified mutation. (**a**) Electropherogram of *ABHD5* exon 6 sequence harboring the c.811G > A mutation in homozygous status in patient, in heterozygous status in parents and compared to a control sequence; (**b**) Secondary structure of ABHD5 (p.G271R) compared to wild-type ABHD5 protein. The p.G271R missense mutation causes modifications of the protein structure at position 171 (from α- helix to β-sheet), 218 (from α- helix to coiled-coil) and 269 (from coiled-coil to α- helix). The alterations are marked by rectangle. p.G271R amino-acid substitution is indicated by arrow; (**c**) Predicted 3D model of ABHD5 and ABHD5 (p.G271R) protein. The p.G271R mutation determines conformational changes of protein folding

Molecular analysis revealed a novel *ABHD5* disease-causing variant (c.811G > A) in homozygous status (Fig. 4a), confirming the patient’s clinical diagnosis. The mutant sequence was submitted to GenBank (accession number MN242826). This pathogenic variant was not found in 100 healthy controls. It causes the substitution of a single amino-acid in position 271 of the ABHD5 protein (p.G271R). Bioinformatic analyses were carried out to evaluate the impact of the p.G271R mutation on ABHD5 function. The alignment of ABHD5 protein sequences in eleven vertebrates revealed that the amino acid G271 is located in a highly conserved site. Moreover, investigation performed by PolyPhen-2 and Mutation Tasting showed that p.G271R mutation probably has a deleterious effect on ABHD5 function. Finally, I-Tasser tool revealed that secondary structure of p.G271R ABHD5 presents conformational changes at positions 171, 218 and 269 (Fig. 4b). These modifications problably correlate with an incorrect 3D folding of the mutant protein (Fig. 4c).

## Discussion

CDS is an autosomal recessive neutral lipid disease that affects the skin, eyes, central nervous system, skeletal muscle, liver and bone marrow. While the extracutaneous manifestations are heterogeneous both in characteristics and in severity, ichthyosiform erythroderma is usually present in all patients, since birth. The typical features of ichthyosis in CDS are extensive scaly lesions on erythematous background [10]. However, the clinical appearance of CDS may, in rare cases, imitate other skin diseases with erythema and scales. Indeed, a case of erythrokeratoderma variabilis-like CDS, presenting patches of normal skin alternating with erythematous scaly patches, has been reported in the literature [11]. The cutaneous lesions of our patient developed at the age of 2 years. Moreover, he presented uninvolved areas between wide plaques with no alternating lesions. In this sense the clinical picture resembled pityriasis rubra pilaris in which the uninvolved areas are the typical feature [12]. The different clinical appearance of skin led to a long delay in reaching the correct diagnosis.

Liver involvement and hiperlipemia are common findings in CDS, occurring in more then 80% of cases. Currently, there is *no* specific *treatment for CDS.* However, *a* diet low in fatty acids with medium chain triglycerides (MCT) supplementation was reported to decrease hepatomegaly and normalize hepatic enzymes, especially when early initiated in combination with vitamin E and ursodeoxycholic acid [4, 7, 13–15]. Although our patient is now 29 years old, after molecular testing, we have recommended a fat-restricted diet+MCT because he presented hepatomegaly, hepatosteatosis and elevation in liver function tests. CK was also elevated in our patient, but myopathy was not detected in EMG and clinically there was no muscle weakness. However, muscle damage has been reported only in 40% of affected subjects [16].

In our CDS family, genetic analysis displayed a novel *ABHD5* missense variant (p.G271R). Bioinformatic investigation, performed with two different predictive tools for missense mutations, showed that glycine to arginine substitution at position 271 may affect protein activity. Moreover, to clarify whether p.G271R can also alter ABHD5 protein folding, tridimensional (3D) structures of native and mutant proteins were generated. The analysis, obtained by I-Tasser software, revealed that the p.G271R substitution causes a modification of the secondary structure of the protein in 3 different regions. These alterations can determine dramatic 3D conformational changes of the ABHD5 protein affecting its ability to bind lipid droplet surface and to interact with proteins involved in neutral lipid metabolism (ATGL/PNPLA2) and skin permeability barrier formation (PNPLA1). To our best knowledge, 37 *ABHD5* different types of mutations have been reported in CDS patients. Most of them cause the production of truncated proteins or determine protein synthesis deficiency, while the missense mutations represent 10% of the pathologic variations^1,12^. Some authors failed to find any genotype-phenotype correlation, not even considering only patients carrying the same mutation, p.N209X, which is the most frequent in CDS [3, 17].

The p.G271R ABHD5 mutation has never been reported previously, but molecular and bioinformatic investigation clearly showed that it represents pathogenic variant correlated to CDS onset. At the moment, there is not sufficient clinical and genetic knowledge to explain why our patient presents with pityriasis rubra pilaris-like erythroderma instead of classical type of congenital ichthyosiform erythroderma. Environmental factors, as UV exposition, medication intake or vaccination, diet and lacking of specific nutritional elements should be taken into account as potentially related to this clinical phenotype.

## Conclusion

In this report, we detected a novel missense *ABHD5* mutation and described an unusual skin manifestation in a patient. Only further genetic investigations on new pityriasis rubra pilaris-like CDS cases will be able to clarify whether the novel variant is associated or not with the distinct type of ichthyosiform skin lesions, expanding the spectrum of clinical phenotype for CDS. As CDS is an extremely rare genetic disorder and its pathophysiology is largely unclear, it is important to emphasize the accurate description of novel patients in order to improve the knowledge of the natural history and explore genotype-phenotype correlation.

## Data Availability

Data obtained during this study are included in the article.

## Abbreviations

CDS: Chanarin Dorfman Syndrome
CK: Creatine kinase
EMG: Electromyographic examination
LD: Lipid droplet
MCT: Medium chain triglycerides
MGG: May-Grünwald-Giemsa
NCIE: Non-bullous congenital ichthyosiform erythroderma
PRP: Pityriasis rubra pilaris
TG: Triacylglycerol
UV: Ultra violet
